# Exploiting the behaviour of wild malaria vectors to achieve high infection with fungal biocontrol agents

**DOI:** 10.1186/1475-2875-11-87

**Published:** 2012-03-26

**Authors:** Ladslaus L Mnyone, Issa N Lyimo, Dickson W Lwetoijera, Monica W Mpingwa, Nuru Nchimbi, Penelope A Hancock, Tanya L Russell, Matthew J Kirby, Willem Takken, Constantianus JM Koenraadt

**Affiliations:** 1 Biomedical and Environmental Group, Ifakara Health Institute, P.O. Box 53, Off Mlabani Passage, Ifakara, Tanzania; 2 Laboratory of Entomology, Wageningen University and Research Centre, P.O. Box 8031, 6700 EH Wageningen, The Netherlands; 3 Pest Management Centre, Sokoine University of Agriculture, P.O. Box 3110, Morogoro, Tanzania; 4 Faculty of Biomedical and Life Sciences, University of Glasgow, 120 University Place, G12 8TA Glasgow, UK; 5 School of Life Sciences, University of Warwick,, Coventry CV4 7AL, UK; 6 The University of Queensland, School of Population Health, Australian Centre for Tropical and International Health, Brisbane 4006, Australia; 7 Vector Group, Liverpool School of Tropical Medicine, Liverpool L3 5QA, UK

## Abstract

**Background:**

Control of mosquitoes that transmit malaria has been the mainstay in the fight against the disease, but alternative methods are required in view of emerging insecticide resistance. Entomopathogenic fungi are candidate alternatives, but to date, few trials have translated the use of these agents to field-based evaluations of their actual impact on mosquito survival and malaria risk. Mineral oil-formulations of the entomopathogenic fungi *Metarhizium anisopliae *and *Beauveria bassiana *were applied using five different techniques that each exploited the behaviour of malaria mosquitoes when entering, host-seeking or resting in experimental huts in a malaria endemic area of rural Tanzania.

**Results:**

Survival of mosquitoes was reduced by 39-57% relative to controls after forcing upward house-entry of mosquitoes through fungus treated baffles attached to the eaves or after application of fungus-treated surfaces around an occupied bed net (bed net strip design). Moreover, 68 to 76% of the treatment mosquitoes showed fungal growth and thus had sufficient contact with fungus treated surfaces. A population dynamic model of malaria-mosquito interactions shows that these infection rates reduce malaria transmission by 75-80% due to the effect of fungal infection on adult mortality alone. The model also demonstrated that even if a high proportion of the mosquitoes exhibits outdoor biting behaviour, malaria transmission was still significantly reduced.

**Conclusions:**

Entomopathogenic fungi strongly affect mosquito survival and have a high predicted impact on malaria transmission. These entomopathogens represent a viable alternative for malaria control, especially if they are used as part of an integrated vector management strategy.

## Background

Currently, insecticide treated nets (ITNs) and indoor residual spraying (IRS) are the mainstay of global efforts towards malaria elimination [1,2]. These measures have proven effective in controlling the disease, but this is threatened by the mosquito vectors developing resistance to the synthetic insecticides [3-6]. For example, assessment of the effect of ITNs in Benin revealed that in areas with insecticide-resistant populations of *Anopheles gambiae*, ITNs no longer prevent such mosquitoes from blood feeding or increase their mortality [7,8]. Similarly, in Senegal, a rebound and age shift in malaria cases was observed following introduction of ITNs and artemisinin combination therapy [9]. Clearly, there is an urgent need to develop novel malaria control strategies that can be reliably and sustainably used to complement or replace existing control measures [10]. Biological control of adult mosquitoes using entomopathogenic fungi offers such an alternative approach. Laboratory and small-scale field trials have demonstrated that malaria vectors can succumb to fungus infection [11-15]. Moreover, mosquitoes resistant to insecticides are still vulnerable to fungal infection, and insecticides and fungi could even work synergistically [16-18]. However, efficient techniques that disseminate fungus at larger scale to populations of wild malaria mosquitoes have not been developed [19].

Host-seeking mosquitoes could be targeted with fungal formulations when entering a house through the eaves [20,21], or when attacking a host under a bed net. Resting mosquitoes could be targeted indoors on walls [22] or both indoors and outdoors by means of point source targets, e.g. resting boxes, clay pots, and black cotton cloths attached to the roof or wall [12,15,23]. Outdoor bait-stations have also demonstrated potential as dissemination tools [13]. In reality, the effectiveness of a particular delivery technique will depend on the behaviour of the locally transmitting malaria vector species. For successful control with entomopathogenic fungus it is not only necessary that the mosquito contacts a treated surface, but also receives a sufficient dose of infectious conidia upon this contact [24,25].

The effectiveness of five different techniques of fungal exposure was examined. Each of these exploited the behaviour of mosquitoes when they were either entering, host-seeking or resting in experimental huts in a highly malaria endemic area in rural Tanzania. As African malaria vectors tend to blood feed and rest primarily indoors [26], fungal delivery techniques focused on exposing mosquitoes to entomopathogenic fungi inside the house. Based on the obtained data on fungal infection rates and virulence, a population dynamic model of mosquito-malaria interactions was implemented to estimate the impact of fungal infection on malaria transmission. In addition, the role of outdoor biting behaviour on the effectiveness of the approach was explored.

## Methods

### Study area

The field trials were conducted in Lupiro village (8.38° S and 36.67° E) (Ulanga District), a rural hamlet 30 km south of Ifakara, in the Kilombero valley of Tanzania. The village lies on a low plateau of about 10 m above the surrounding area at an altitude of 300 m above sea level. The area borders a permanent swamp (near the Ndolo River) extensively cleared for rice cultivation. Most of the residents are farmers and in addition to rice they cultivate maize and cassava. The majority of the houses are made from mud walls with thatched roofs. There are two rainy seasons: the long rains from April to June and short rains normally in October and November. The annual rainfall is about 1200 - 1800 mm. The temperature ranges between 20°C and 32.6°C. The present study was conducted between May and December 2009 (trials 1-4) as well as between March and April 2010 (trial 5). The population of malaria vectors in the area is largely comprised of members of the *An. gambiae *complex, mainly *Anopheles arabiensis *(98%) and few *An. gambiae *sensu stricto [27]. *Anopheles funestus *occurs at low densities. Estimates of the entomological inoculation rate indicate that people receive an estimated 81 infectious bites per year [28].

### Entomopathogenic fungus species and formulation

Two fungal species, *Metarhizium anisopliae *var. *anisopliae *(isolates ICIPE-30 and IP 46 [29]) and *Beauveria bassiana *(isolate: I93-825 (IMI 391510)) were used in the trials. The species and strains of fungus actually used in each trial depended on availability (see Experimental hut trials). Fungal conidia were suspended in a 1: 1 mixture of highly refined Enerpar oil (Enerpar M002^®^, BP Southern Africa Ltd) and Shellsol oil (Shellsol T^®^, South Africa Chemicals) [30]. Except for the netting materials in trial 1 (left to dry in the shade for 5 h), cloth materials were left to dry indoors for 72 h at ambient temperature. Unless stated otherwise, treatment of materials was done at the laboratories of the Ifakara Health Institute (30 km from the study site), and the materials were transported to the field site after drying. Before application in the trial, viability of conidia was confirmed by inoculation on Sabouraud dextrose agar (SDA). The percentage germination of conidia used for the first three trials ranged between 80 - 85% and that of the conidia used for the last two trials ranged between 70 - 75%.

### Experimental huts

**Figure 1:**
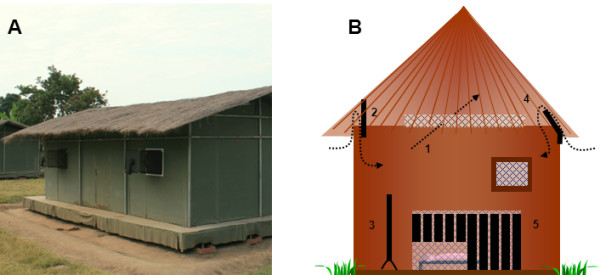
**A: picture of experimental hut in Lupiro, Tanzania; B: schematic drawing of the five designs evaluated for infecting malaria vectors with entomopathogenic fungi inside the experimental huts**. 1: eave netting, 2: cotton cloth eave curtain, 3: cotton cloth panel, 4: cotton cloth eave baffle and 5: cotton cloth strips around bed net.

The experimental huts were designed to represent local housing [31]. The roof consisted of corrugated iron lined with thatch. The outer walls were constructed from canvas. Inner walls were made of removable panels coated with mud, to simulate local mud walls. The huts were 6.5 m long, 3.5 m wide and 2 m high. The height of each structure measured 2.5 m at the roof apex. Three huts were used in the trials and each hut had 4 windows all fitted with exit traps (Figure 1A). The huts were positioned between the village and a nearby rice field, standing approximately 15 m apart. In 2009, temperature and relative humidity inside the experimental huts ranged from 13.8 - 37.9°C and 30.3 - 100% RH, respectively. For the experimental months in 2010, temperature and relative humidity ranged from 22.5 - 37.7°C and 45.0 - 99.8% RH, respectively.

### Experimental hut trials

Three experimental huts were used in each of five trials. Each individual trial was conducted for nine days. Unless stated otherwise, a 3 × 3 Latin square design was used to simultaneously evaluate two different designs for delivering entomopathogenic fungi and one control. Treatments were randomly allocated to the three different huts, and were switched between huts each time after three days. During the trials each hut contained two human volunteers sleeping under untreated bed nets. The volunteers rotated between huts daily such that each pair spent three days in each hut in total. The volunteers slept in the experimental huts from 19.00 - 7.00 h. Exit traps were installed over all four windows (trap: 55.5 × 45.5 × 55.5 cm; funnel opening: 40 × 3 cm) to catch mosquitoes when they exit, except for trial 1 where two window and six eave traps (trap: 100 × 40 × 40 cm; funnel opening: 80 × 3 cm) were used. Mosquitoes were collected by mouth aspirator from the exit traps at 7.00 am in the morning. A random sub-sample of maximum 25 female mosquitoes from each hut was collected. Each mosquito was placed individually into 50 ml falcon tubes (115 mm × 28 mm diam.) that were covered with gauze and provided with sugar water-soaked cotton-wool on top. Daily survival of mosquitoes was monitored in a field-based insectary until all mosquitoes had died (maximum time to death: 39 days). When less than 25 mosquitoes were trapped in the window exit traps, all available female individuals were used. Mosquitoes remaining in the traps were killed, identified and stored. Cadavers of mosquitoes that died in the survival experiment were left to dry in open air for 2 day, put onto filter paper in Petri dishes covered with their lids and kept inside transparent containers (30 cm diameter and 40 cm high) with wet towels (humid chambers) for 5-6 day. Thereafter, they were examined for fungal sporulation. Containers were tightly closed with lids to maintain humidity.

### Trial 1: Eave netting

Fungus-treated polyester netting (9 holes/cm^2^) was fitted over the eaves of the experimental huts to determine whether wild mosquitoes could be infected during passage through this netting (Figure 1-B). In the laboratory, 40% of the mosquitoes passed through this type of netting when exposed to a human stimulus. A fungal formulation of *Beauveria bassiana *I93-825 was painted with a brush onto the netting laid on a flat surface at a concentration of 2 × 10^10 ^conidia/m^2^. Treated netting was left outdoors in the shade to dry for about 5 h, before being fitted over the eaves with Velcro strips. Two controls were used: eaves with oil-treated netting, and eaves without netting.

### Trial 2: Eave curtain

Black cotton curtains (20 cm high) were fixed from the top with Velcro strips and left hanging in front of the eaves, leaving a gap of about 3.5 cm between the cloth and the wall (Figure 1B). Mosquitoes passing the eaves could fly into the hut through the gap at the lower end of the curtain, probably after contacting the curtain to locate the gap. One hut had curtain treated with oil alone (control), and the two other huts had curtains treated with *B. bassiana *I93-825 or *M. anisopliae *ICIPE-30, respectively. Both isolates were applied onto curtains at a concentration of 2 × 10^10 ^conidia/m^2 ^using procedures described by Mnyone et al. [14]. Only the surface of the curtain facing the outside was treated.

### Trial 3: Eave curtains and panels

Eave curtains were placed in one of the huts, as in Trial 2, except that a four times higher concentration of fungus (8 × 10^10 ^conidia/m^2^) was used. In the other hut, two black cotton panels (length 120 cm, width 90 cm), one per bed were used. Panels were treated with fungus on both sides by the use of a hand sprayer [14], and were placed next to the bed facing the feet of a sleeping volunteer (Figure 1B). Curtains and panels were both treated with *B. bassiana*. The control hut had neither curtain nor panel and eaves were left open.

### Trial 4: Eave baffles

Eave curtains were modified to serve as eave baffles [32]. Unlike curtains that were parallel to the wall and had the entry gap at the lower side, cotton cloth baffles were fitted in a slanting orientation with the entry gap (3 cm) from the top (≈ 20 cm inside the wall) (Figure 1B). At the bottom, the cloth material was fixed with pins to the outside wall covering ≈ 10 cm of the outside wall surface. One hut had baffles treated with *Beauveria bassiana *I93-825 and one had baffles treated with *M. anisopliae *IP 46 at a concentration of 4.1 × 10^10 ^conidia/m^2^. The control hut had eave baffles treated with oil alone.

### Trial 5: Bed net strips

Long black cotton strips (126 cm, 7 cm wide) and short black cotton strips (63 cm, 7 cm wide) were treated on one side with *M. anisopliae *IP 46 at a concentration of 5 × 10^10 ^conidia m^-2 ^and installed next to bed nets to surround the entire bed (Figure 1). The gap between the strips was 1 cm. One hut had short cloth strips, one hut long cloth strips and the third hut did not have any strips (control). Treatment of the strips was done at the field site at 17: 00 h to minimize the effect of sun light on conidia when drying. Strips were left outdoors for 3 h to allow initial drying, then installed inside huts and left for an extra 24 h to complete drying.

### Data analysis and modelling

Weibull functions representing proportional survival of adult mosquitoes as a function of time were fitted to the survival data of the daily-collected mosquitoes from the experimental huts (see Additional file 1 and [33]). These functions were used to estimate the average time to death for mosquitoes collected from control and treatment huts for each trial. These estimates, along with the observed daily fungal infection rates, were used to parameterize a population dynamic model developed by of one of us (PAH) [33] to estimate the impact of fungus application on malaria transmission rate, expressed as the daily entomological inoculation rate (EIR) [34]. The model incorporates details of mosquito life history relevant to the effect of fungal biopesticide interventions on malaria transmission, including time-dependent adult mortality, gonotrophic feeding processes (host-seeking and non-host-seeking mosquitoes) and insecticide resistance. Unless otherwise specified the same parameter values as those in Hancock (2009) were used [33].

The coverage of the biopesticide was defined as the daily probability that host-seeking mosquitoes contract the fungus, and assume that non-host-seeking mosquitoes are not at risk of fungal infection [33]. The fitted Weibull functions were used to estimate the effect of fungal infection on adult mosquito mortality in the absence of all other mortality sources (see Additional file 1). The average time until death from fungal infection excluding all other mortality sources is used to summarize the effect of infection on mosquito mortality and can be considered a measure of the 'virulence' of the fungal biopesticide.

### Modelling the impact of biting behaviour

The model also allows for the incorporation of fine-scale temporal variation in biting behaviour. The goal of this exercise was to account for outdoor biting patterns of *An. arabiensis *populations at the study site ([35]). Given that data are not available to accurately quantify biting behaviour and its implications for fungal infection risk, a conservative set of assumptions are adopted. Parameters relating to indoor and outdoor biting behaviour are given in Table 1 and a more detailed explanation of the full model and its parameterization is given in Additional file 2. In summary, the mosquito population is divided into two subpopulations that display different levels of indoor and outdoor biting behaviour. The first subpopulation is endophilic and seeks blood meals indoors when humans are indoors. The fungal infection risk for this subpopulation is parameterized using data from the experimental hut trials. The second subpopulation is exophilic and seeks hosts outdoors for most of the time that humans are indoors. Both subpopulations seek human blood meals during times that humans are outdoors, during which time mosquitoes are assumed to be not exposed to the biopesticide. The endophilic subpopulation takes blood meals only from humans while the exophilic subpopulation may take human or non-human blood meals. Therefore, while the endophilic subpopulation is responsible for greater levels of malaria transmission, it is also exposed to a greater risk of fungal infection.

**Table 1:** Parameter definitions and assumed values of the model of Hancock et al.

Symbol	Definition	Value	Source
*f*	Rate of finding blood meals for host-seeking mosquitoes	2.4 (d^-1^)	[36]
*P*	Probability of finding a blood meal for host-seeking mosquitoes within a 12 hour host-seeking period, given that they do not die (1-exp(- *T_H _f*))	0.7	
*p_E_*	Proportion of the total mosquito population that comprise the exophillic subpopulation	0.58	[35]
Q	Probability that exophillic mosquitoes feed on a human host	1/3	[37]
*T_H_*	Duration of the host-seeking period	0.5 (d)	[35]
*p_o_*	Proportion of the host-seeking period during which humans are outdoors	0.2	[35]
*F*	Rate of contracting fungal infection for endophilic and exophilic subpopulations	3.0, 3.0/5 (d^-1^)	This study
*C*	Probability of fungal infection during the period of biopesticide exposure for endophilic and exophilic subpopulations (1-exp(-(1-*p_o_*)*T_H _F*))	0.7, 0.2	This study

[34]

### Ethical approval

The study was conducted after being approved by the Institutional Review Board of the Ifakara Health Institute (IHI) (IHRDC/IRB/No. A-019) and the National Institute of Medical Research (NIMR/HQ/R.8a/Vol. IX/710) in Tanzania.

## Results

### Experimental hut trials

The netting prevented females from entering, but failed to infect them with fungus (trial 1; Table 2). Treated eave curtains and cloth panels also failed to infect mosquitoes with either *M. anisopliae *or *B. bassiana*. Although the increased dosage in trial 3 resulted in 11-18% of mosquitoes being infected with *B. bassiana*, exposure to entomopathogenic fungi only resulted in slightly elevated mortality (trials 2 and 3; Table 2; see Additional files 3 and 4 for estimated model parameters).

**Table 2:** Number of female *Anopheles gambiae *s.l. collected nightly from each treatment of the five experimental hut trials, the proportion that showed fungal growth after death (Sporulation %) and the average time to death (*g*) derived from the Weibull models in days (see Additional file 1)

						Avg. time to death (*g*)		
Trial	Design	Treatment	Dosage (conidia/m^2^)	Average catch per night	Sporulation (%)	All	Uninfected	Infected
1	Eave netting	*Bb *I93-825	2*10^10^	1.1 ± 0.35	0.0	nd		
		Oil control		1.0 ± 0.24	0.0	nd		
		Open eaves (control)		45.0 ± 7.8	0.0	nd		
2	Eave curtain	*Ma *ICIPE-30	2*10^10^	36.4 ± 6.0	0.0	15.6		
		*Bb *I93-825	2*10^10^	32.4 ± 4.4	0.0	17.2		
		Oil control		38.4 ± 4.1	0.0	18.4		
3	Eave curtain	*Bb *I93-825 on curtain	8*10^10^	27.9 ± 1.7	18.3	18.5		
	& panels	*Bb *I93-825 on panels	8*10^10^	30.2 ± 1.3	10.7	17.3		
		Open eaves (control)		33.9 ± 1.2	0.0	20.6		
4	Eave baffles	*Ma *IP 46	4.1*10^10^	40.6 ± 8	69.1		20.7	11.5
		*Bb *I93-825	4.1*10^10^	56.3 ± 11.4	67.9		17.9	10.5
		Oil control		74.2 ± 14.5	2.0		20.3	
5	Bed net strips	*Ma *IP 46 - long	5*10^10^	24.3 ± 3.2	75.5		24.5	10.5
		*Ma *IP 46 - short	5*10^10^	38.4 ± 6.8	74.6		23.0	11.8
		Open eaves (control)		86.2 ± 12.7	3.3		19.2	

As sporulation rates in the trials 2 and 3 were zero or very low, we only calculated separate 'average time to death' for fungus-infected individuals (those that sporulated) and fungus-uninfected individuals (those that did not sporulate) of trials 4 and 5. In trial 1, the number of mosquitoes collected in exit traps of treatment huts was too low to calculate 'average time to death' ('nd' in Table). *Ma*: *Metarhizium anisopliae*; *Bb*: *Beauveria bassiana*

**Figure 2:**
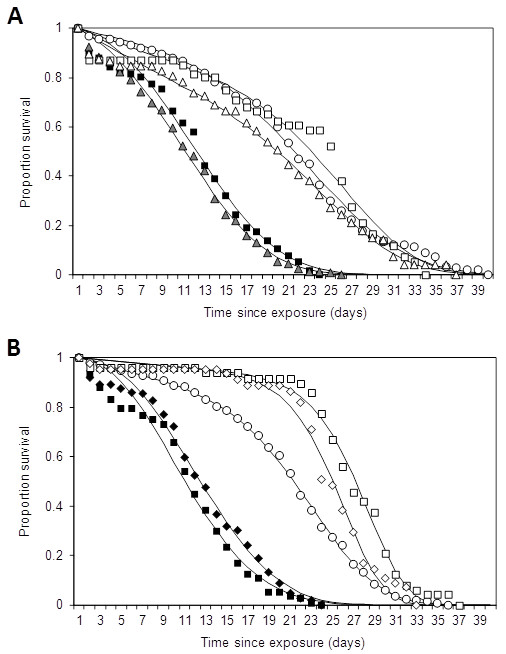
**Survival of wild female *Anopheles gambiae *s.l. mosquitoes collected from experimental huts**. A: Results from trial 4 (eave baffles) with white circles: control; white squares: uninfected females from *Metarhizium anisopliae *IP 46 treated huts; black squares: infected females from *M. anisopliae *IP 46 treated huts; white triangles: uninfected females from *Beauveria bassiana *I93-825 treated huts; gray triangles: infected females from *B. bassiana *I93-825 treated huts. B: Results from trial 5 (cloth strips treated with *M. anisopliae *IP-46 around bed net) with white circles: control; white diamonds: uninfected females from short strips in the treated hut, black diamonds: infected females from short strips in treated hut; white squares: uninfected females from long strips in the treated hut; black squares: infected females from long strips in the treated hut. Solid lines show Weibull functions fitted to each survival profile using the least squares method (see Additional file 1).

In trial 4 (eave baffles), the average number of female *An. gambiae *mosquitoes collected per hut per night ranged from 40.6 to 74.2 (Table 2). Of 178 mosquitoes that were sampled from the *M. anisopliae *IP 46 treated hut, 69% showed fungal growth. For the *B. bassiana *treated hut, 68% of 206 mosquitoes showed fungal growth (Table 2). Of the controls, 2.0% showed fungal growth. Mosquitoes from huts with fungus-treated baffles survived less long relative to mosquitoes collected from the control hut, regardless of the fungal species (Table 2; Figure 2-A; see Additional files 5 and 6 for estimated model parameters). Average time to death of fungus-infected mosquitoes was reduced by 43% (*M. anisopliae*) and 38% (*B. bassiana) *compared to mosquitoes collected from control huts. It was reduced by 44*% *(*M. anisopliae*) and 41% *(B. bassiana*) compared to uninfected females collected from the same treatment.

In trial 5 (bed net strips), the average number of female *An. gambiae *mosquitoes collected per hut per night ranged from 24.3 to 86.2 (Table 2). Of 155 mosquitoes sampled from huts with fungus-treated long strips, 75.5% showed fungal growth. From huts with treated short strips, 74.6% of 189 mosquitoes showed fungal growth. Of the controls, 3.3% showed growth of *M. anisopliae *(Table 2). Survival of mosquitoes from huts with both long and short strips was substantially lower compared to mosquitoes collected from the control (Table 2; Figure 2-B; see Additional files 7 and 8 for estimated model parameters): average time to death of fungus-infected mosquitoes was reduced by 45% (long strips) and 39% (short strips) compared to mosquitoes collected from control huts. It was reduced by 57*% *and 49%, respectively, if compared to uninfected females collected from the same treatment hut.

### Impact of entomopathogenic fungi on EIR

**Figure 3:**
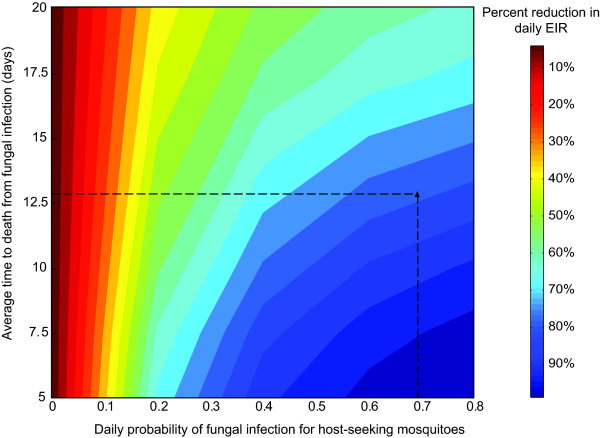
**The model-estimated percent reduction in daily EIR for varying levels of the average time to death from fungal infection (fungal virulence) and the daily probability of fungal infection for host-seeking mosquitoes (fungal biopesticide coverage)**. Dashed lines show the coverage and the effect of fungal infection on mortality reported for the trial of *Beauveria bassiana *I93-825 on eave-baffles (Figure 2A).

Assuming the most successful delivery methods are employed (eave baffles and bed net strips; trials 4 and 5), a similar impact of *M. anisopliae *and *B. bassiana *on malaria transmission can be expected because the experiments using both isolates produced similar reductions in survival (Figure 2). Therefore, only the survival data of mosquitoes exposed to *B. bassiana *applied on eave baffles (trial 4; Figure 2A) were used to estimate the parameters of the Weibull function describing the time-dependent effect of fungal infection on mosquito mortality. Based on fungal infection rates obtained from the experiment, the daily probability of fungal infection for host-seeking mosquitoes, or the fungal coverage, is estimated to be 0.68. For these levels of coverage and effects on mortality, the estimated reduction in the daily EIR that could be achieved by our *B. bassiana *application is 75-80% (Figure 3).

### Impact of feeding behaviour

With a fungus coverage level as observed in the present study (0.7), the efficacy of the fungus is not greatly reduced when approximately 58% of the total population displays exophilic biting behaviour (Russell et al., 2011, Figure 4). Similar conclusions can be drawn when coverage levels are lower, provided that the coverage is not at very low levels e.g. below 0.2 (see Additional file 9). In general, only when the whole population starts displaying exophilic behaviour, the effectiveness of the indoor intervention is substantially reduced.

**Figure 4:**
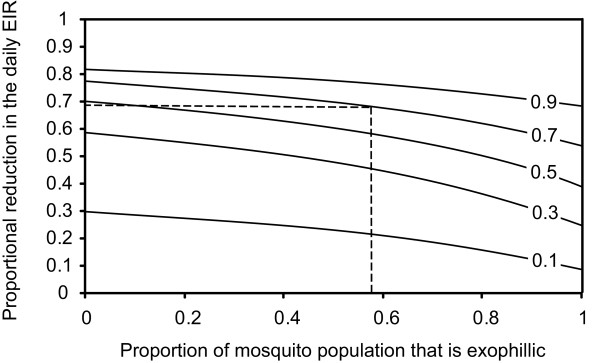
**The proportional reduction in the daily EIR as a function of the proportion of the mosquito population that is exophilic**. Line labels show the fungal biopesticide coverage. Dotted lines show the reduction in EIR for the biopesticide coverage value measured in the experimental hut trials (this study) and the estimated proportion of the *An. arabiensis *population that is exophilic for the study area [35].

## Discussion

Up to three quarters of house-entering mosquitoes became infected with entomopathogenic fungi when either the eaves were provided with fungus-treated baffles or when strips of fungus-treated cotton cloth were hung around the bed net. This infection resulted in an increased daily risk of death and, according to the model, such coverage and virulence rates lead to an estimated 75-80% reduction in malaria transmission. By contrast, eave netting, eave curtains and cotton panels placed next to the bed were ineffective in exposing mosquitoes to fungi and did not affect survival or even infect mosquitoes with fungal spores. Mosquitoes which contacted fungus-treated netting (trial 1) and flew off may have become infected, but this could not be confirmed with the experimental design that was used. Conidia could lose their potency rapidly when applied on netting [38,39]. With eave curtains made of cotton cloth (trial 2), mosquitoes possibly flew directly into the huts without contacting the treated surfaces or spent too little time on the cloth to pick up conidia [24,25]. This may result in small infective doses that can be countered by immune responses such as melanisation, encapsulation and phagocytosis [40]. Although the concentration of fungal conidia in the subsequent trial (trial 3) was four times higher, the impact of the fungus was still small with fungus infection rates reaching 11 and 18% for curtains and panels, respectively.

Quite strikingly, in huts with baffles that were fitted in a slanted orientation with the entry gap at the top (trial 4), mosquito survival was almost halved. Apparently, a minor modification of the position of fungus-treated surfaces greatly increased the probability of mosquitoes contacting, and spending time on treated surfaces. Similarly, after entering the house, the majority of host-seeking mosquitoes could be targeted when approaching a bed net as demonstrated with the cotton cloth strips hung next to the net. For such fungal tools to have a strong effect on the EIR, it has been estimated that > 50% of house entering mosquitoes would need to be infected and their survival reduced below the critical age at which they can pass on the malaria parasite [15,41]; in the present study a fungal coverage as high as 75% was achieved. With this coverage level, a more virulent fungus (i.e. with lower time to death) will only marginally increase effectiveness in terms of a further reduction in EIR (see Figure 3; moving down the vertical dashed line). When only a low proportion of the mosquito population comes into contact with the fungus, virulence becomes more important. For example, at a coverage level of 0.3 and a virulence of 12.5 days, the reduction in EIR is ~55%. A virulence of ~6 days would be required to achieve a similar reduction of 80% in EIR. Below coverage levels of 0.2, even highly virulent strains will not achieve major reductions in transmission. Similarly, with the currently achieved coverage, even fungus with relatively low virulence (slow kill), e.g. as a result of natural degradation, can still produce substantial reductions in the EIR (see Figure 3; moving up the vertical dashed line).

The first model estimates presented in this paper are based on the numbers of (mostly *An. arabiensis*) mosquitoes actually in search for a human host sleeping inside the experimental huts at night. This is the time and location where most of the transmission takes place. However, because ITNs may select for outdoor-biting behaviour within or between species [35,42], the impact of exophily on the effectiveness of the biopesticide approach was explored as well. This model analysis showed that the presence of considerable outdoor feeding activity does not significantly impact on the ability of indoor biopesticide interventions to reduce the EIR. A similar conclusion was reached by modelling analysis which explored the impact of outdoor biting behaviour on the efficacy of indoor ITN interventions [43]. One way outdoor feeding could be addressed is by also targeting these populations with entomopathogenic fungi, e.g. through the use of fungus treated resting stations [13].

Surprisingly, a small proportion (≤ 3%) of mosquitoes from control huts showed fungal growth (trials 4 and 5). This could be explained by assuming that few mosquitoes remained undetected in the huts during the removal of mosquitoes prior to switching the treatments or that mosquitoes from treatment huts visited control huts during the same night or later. In trial 4 and 5, the average number of female anopheline mosquitoes collected from control huts was higher than that collected from treatment huts. As treatments and volunteers were rotated among huts, this cannot have been due to a positional effect or individual variation in attractiveness. This difference could be due to a behavioural effect of *M. anisopliae *IP 46, causing repellency or avoidance, but such effects have not been observed with *M. anisopliae *ICIPE-30 and *B. bassiana *I93-825 in laboratory studies [44]. The behaviour of mosquitoes once infected with a fungus, the sub-lethal consequences of fungal infection [45] and the potential repellency of fungus treated material need to be further investigated under field conditions.

The effectiveness of the bed net strip design demonstrates that it is feasible to develop mosquito control strategies based on lure and kill principles. In the case presented here, the protected sleeper acts as the lure, while the strips delivered the lethal doses of entomopathogenic fungi. Because of their focal nature, such strategies are likely to be more cost-effective than residual application of fungus formulations. In addition, entomopathogenic fungi are slow-killing agents, but kill the mosquito before they are capable of transmitting the infective sporozoite stages. This reduces the pressure for selection of resistance, making these agents 'evolution-proof' and thereby enhancing their long-term use [46].

## Conclusions

The results obtained in realistic field settings provide a necessary stepping stone towards scaling up of fungal biocontrol agents to whole village application. They represent a viable alternative for malaria control, especially if they are used as part of an integrated vector management strategy. Efforts geared at producing high quality fungal products in terms of virulence and persistence should be continued as there is an extra benefit to be accrued in terms of their impact on malaria transmission and the sustainability of malaria control programmes.

## Competing interests

All authors have no conflicts of interest to declare and all have actively contributed to this study and review.

## Authors' contributions

Conceived and designed the experiments: LLM, INL, MJK, WT. Performed the experiments: LLM, MWM, NN, INL, DWL. Analysed the data: LLM, CJMK, INL. Performed the modelling: PAH. Wrote the paper: LLM, CJMK, WT, TLR. Reviewed the paper: CJMK, MJK, WT. All authors read and approved the final manuscript.

## Supplementary Material

Additional file 1 **Explanation of the methods developed by Hancock et al**. (2009) to model adult mosquito survival and evaluate the impact of entomopathogenic fungi on mosquito mortality and malaria transmission. Click here for file

Additional file 2 **Explanation of assumptions in relation to the model that assesses the effect of outdoor feeding on the efficacy of indoor-based fungal applications on EIR**. Click here for file

Additional file 3 **Table S1 Parameters of the model of mosquito mortality estimated from experimental data of trial 2: curtains treated with *Beauveria bassiana *and *Metarhizium anisopliae***. Parameter values were chosen to minimise the residual sum of squares. *μ *is the mortality rate (per day), *β_s _*and *r_s _*are the dimensionless shape and rate shape parameters of the Weibull function, respectively, and *g *is the average time to death (in days) estimated from the Weibull function (see Additional file 1). Click here for file

Additional file 4 **Table S2 Parameters of the model of mosquito mortality estimated from experimental data of trial 3: eave curtains and panels treated with *Beauveria bassiana***. Parameter values were chosen to minimise the residual sum of squares. *μ *is the mortality rate (per day), *β_s _*and *r_s _*are the dimensionless shape and rate shape parameters of the Weibull function, respectively, and *g *is the average time to death (in days) estimated from the Weibull function (see Additional file 1). Click here for file

Additional file 5 **Table S3 Parameters of the model of mosquito mortality estimated from experimental data of trial 4: eave baffles treated with *Metarhizium anisopliae***. Parameter values were chosen to minimise the residual sum of squares. Separate models were fitted for mosquitoes infected and uninfected with the fungus. *μ *is the mortality rate (per day), *β*, *β_s _*and *r*, *r_s _*are the dimensionless shape and rate shape parameters of the Weibull functions, respectively. *g *is the average time to death (in days) estimated from the Weibull function and *g_F _*is the estimated average time to death from the fungus infection alone (see Additional file 1). Click here for file

Additional file 6 **Table S4 Parameters of the model of mosquito mortality estimated from experimental data of trial 4: eave baffles treated with *Beauveria bassiana***. Parameter values were chosen to minimise the residual sum of squares. Separate models were fitted for mosquitoes infected and uninfected with the fungus. Separate models were fitted for mosquitoes infected and uninfected with the fungus. *μ *is the mortality rate (per day), *β*, *β_s _*and *r*, *r_s _*are the dimensionless shape and rate shape parameters of the Weibull functions, respectively. *g *is the average time to death (in days) estimated from the Weibull function and *g_F _*is the estimated average time to death from the fungus infection alone (see Additional file 1). Click here for file

Additional file 7 **Table S5 Parameters of the model of mosquito mortality estimated from experimental data of trial 5: long strips treated with *Metarhizium anisopliae***. Parameter values were chosen to minimise the residual sum of squares. Separate models were fitted for mosquitoes infected and uninfected with the fungus. Separate models were fitted for mosquitoes infected and uninfected with the fungus. *μ *is the mortality rate (per day), *β*, *β_s _*and *r*, *r_s _*are the dimensionless shape and rate shape parameters of the Weibull functions, respectively. *g *is the average time to death (in days) estimated from the Weibull function and *g_F _*is the estimated average time to death from the fungus infection alone (see Additional file 1). Click here for file

Additional file 8 **Table S6 Parameters of the model of mosquito mortality estimated from experimental data of trial 5: short strips treated with *Metarhizium anisopliae***. Parameter values were chosen to minimise the residual sum of squares. Separate models were fitted for mosquitoes infected and uninfected with the fungus. Separate models were fitted for mosquitoes infected and uninfected with the fungus. *μ *is the mortality rate (per day), *β*, *β_s _*and *r*, *r_s _*are the dimensionless shape and rate shape parameters of the Weibull functions, respectively. *g *is the average time to death (in days) estimated from the Weibull function and *g_F _*is the estimated average time to death from the fungus infection alone (see Additional file 1). Click here for file

Additional file 9 **Daily EIR as a function of the fungal biopesticide coverage**. Line labels show the proportion of the mosquito population that is exophilic. Click here for file
