# Supplementary material for: Exploiting the behaviour of wild malaria vectors to achieve high infection with fungal biocontrol agents
Source: Malar J. 2012 Mar 26;11:87. doi: 10.1186/1475-2875-11-87 (PMC3337815; doi:10.1186/1475-2875-11-87)
Supplement: Additional file 1 — Explanation of the methods developed by Hancock et al. (2009) to model adult mosquito survival and evaluate the impact of entomopathogenic fungi on mosquito mortality and malaria transmission. [file 1475-2875-11-87-S1.DOCX]

**Modelling­­ adult mosquito survival**

The methods developed by Hancock et al. (2009) were used to model adult mosquito survival. The model assumes that adult mosquito mortality occurs at a constant daily rate$\mu$. Mosquitoes infected with the fungus are assumed to experience additional mortality at a rate that depends on fungal infection age *u.* This additional mortality is modelled by a Weibull function, so that the mortality rate of fungus-infected mosquitoes, $\mu_{F}(u)$is given by

$\mu_{F}(t)=\mu+\beta r(ru)^{\beta-1}$ (1)

where $\beta$and $r$are the Weibull shape and rate parameters respectively.

*Estimating mortality rates from experimental data*

Data from the experimental hut trials show that the mortality rate of the captured mosquitoes increased over time, whether or not they were infected with the fungus. This is presumably due to a process of senescence. To include senescence in the model of adult mortality, a Weibull function was used to represent the increase in mortality over time since the first sample *t*. Thus mosquitoes uninfected with the fungus experience mortality at a daily rate

$\mu_{U}(t)=\mu+\beta_{s}r_{s}(r_{s}t)^{\beta_{s}-1}$ (2)

where $\beta_{s}$and $r_{s}$are the shape and rate parameters of the Weibull function describing the increase in mortality with time *t*. Mosquitoes infected with the fungus then experience mortality at a rate

$\mu_{F}(t)=\mu+\beta_{s}r_{s}(r_{s}t)^{\beta_{s}-1}+\beta r(ru)^{\beta-1}$ (3)

Parameters $\mu$,$\beta$,$r$, $\beta_{s}$, and $r_{s}$ are chosen to give the best fit to the data in the least squares sense. Table S1 shows the fitted values for each experiment. Figure 2 (main text) shows the actual fitted curves. The fitted model is then used to determine the average time to death following the start of the experiment, *g*, and the average time to death from fungal infection excluding other mortality sources, *g_F_*, as described in Hancock et al. (2009).

Although it is necessary to include senescence to accurately fit the experimental data and estimate the effect of fungal infection on mosquito mortality, mosquitoes in nature are likely to experience a much higher rate of age-independent mortality $\mu$due to predation, swatting by humans and other hosts, and more challenging environmental conditions. Senescence is therefore expected to play a much smaller role in the mortality of wild mosquitoes. Therefore the model that estimates the effect of fungal biopesticide interventions on the EIR does not include the extra senescence term, and considers the mortality of mosquitoes uninfected with the fungus to be governed by the constant rate$\mu$, which is set to a rate observed for field populations of *Anopheles* mosquitoes (see Table 1 of Hancock 2009). The mortality of fungus-infected mosquitoes is estimated by equation (1), where parameters $\beta$,$r$ are estimated from the experimental hut data.
