# Supplementary material for: Exploiting the behaviour of wild malaria vectors to achieve high infection with fungal biocontrol agents
Source: Malar J. 2012 Mar 26;11:87. doi: 10.1186/1475-2875-11-87 (PMC3337815; doi:10.1186/1475-2875-11-87)
Supplement: Additional file 2 — Explanation of assumptions in relation to the model that assesses the effect of outdoor feeding on the efficacy of indoor-based fungal applications on EIR. [file 1475-2875-11-87-S2.DOC]

***Modelling outdoor feeding behaviour***

Fort this modelling study our estimates of the effects of fungal biopesticides on the Entomological Inoculation Rate (EIR) do not consider the presence of insecticide-treated bednets (ITNs), and are based on the specific case of the Hancock (2009) model that the ITN coverage is zero. We also make the conservative assumption that fungal infection does not affect blood feeding activity in adult mosquitoes, which corresponds to the specific case of the Hancock (2009) model for which the rate of finding hosts for host-seeking mosquitoes, *f*, is the same for mosquitoes that are infected and uninfected with the fungus. These assumptions are considered reasonable given that the effects of both the presence of ITNs and reduced blood feeding activity in fungus-infected mosquitoes on the efficacy of the fungal biopesticide in reducing the EIR has been explored by Hancock (2009).

We modified the approach taken by Hancock (2009) by dividing the adult mosquito population into exophillic and endophillic classes. This involves implementing the Hancock (2009) model for two sets of parameters corresponding to endophillic and exophillic behaviour, thus estimating the contribution of endophillic and exophillic subpopulations to the EIR, with the total EIR being the sum of the two contributions. This assumes that individual mosquitoes do not change their degree of endophillic (or exophillic) behaviour throughout their life.

In a study of adult mosquito biting behaviour conducted in southern Tanzania, Russell et al. (2011) estimated that a proportion 0.575 of the (predominantly *Anopheles* *arabiensis*) mosquito population were caught outdoors in human landing catch experiments.  In this study we assume that the 'endophillic' subpopulation spends 20% of the host-seeking period outdoors and the 'exophillic' population spends 85% of the host-seeking period outdoors. We assume that endophillic mosquitoes are host-seeking indoors for the proportion of the host-seeking period during which humans are indoors, which is estimated to be 80% of the host-seeking period (Russell et al. 2011). Exophillic mosquitoes spend 80% less time indoors than endophillic mosquitoes, and so the fungal infection rate experienced by exophillic mosquitoes during the period of biopesticide exposure is estimated to be one fifth of that experienced by endophillic mosquitoes. Table 1 (main text) shows the parameters of the model that are different from those used by the original model presented in Hancock (2009).
