# Supplementary material for: Exploiting the behaviour of wild malaria vectors to achieve high infection with fungal biocontrol agents
Source: Malar J. 2012 Mar 26;11:87. doi: 10.1186/1475-2875-11-87 (PMC3337815; doi:10.1186/1475-2875-11-87)
Supplement: Additional file 3 — Table S1 Parameters of the model of mosquito mortality estimated from experimental data of trial 2: curtains treated with Beauveria bassiana and Metarhizium anisopliae. Parameter values were chosen to minimise the residual sum of squares. μ is the mortality rate (per day), βs and rs are the dimensionless shape and rate shape parameters of the Weibull function, respectively, and g is the average time to death (in days) estimated from the Weibull function (see Additional file 1). [file 1475-2875-11-87-S3.DOCX]

**Table S1.** Parameters of the model of mosquito mortality estimated from experimental data of trial 2: curtains treated with *Beauveria bassiana* and *Metarhizium anisopliae.* Parameter values were chosen to minimise the residual sum of squares. *µ* is the mortality rate (per day), *β_s_* and *r_s_* are the dimensionless shape and rate shape parameters of the Weibull function, respectively, and *g* is the average time to death (in days) estimated from the Weibull function (see Additional file 1).

| Parameter | Control | *Beauveria bassiana* | *Metarhizium anisopliae* |
| --- | --- | --- | --- |
| $\mu$ | 0.0 | 0.0 | 0.017 |
| $\beta_{s}$ | 1.8 | 1.6 | 2.0 |
| $r_{s}$ | 0.048 | 0.052 | 0.047 |
| *g* | 18.4 | 17.2 | 15.6 |
