# Supplementary material for: Exploiting the behaviour of wild malaria vectors to achieve high infection with fungal biocontrol agents
Source: Malar J. 2012 Mar 26;11:87. doi: 10.1186/1475-2875-11-87 (PMC3337815; doi:10.1186/1475-2875-11-87)
Supplement: Additional file 4 — Table S2 Parameters of the model of mosquito mortality estimated from experimental data of trial 3: eave curtains and panels treated with Beauveria bassiana. Parameter values were chosen to minimise the residual sum of squares. μ is the mortality rate (per day), βs and rs are the dimensionless shape and rate shape parameters of the Weibull function, respectively, and g is the average time to death (in days) estimated from the Weibull function (see Additional file 1). [file 1475-2875-11-87-S4.DOCX]

**Table S2.** Parameters of the model of mosquito mortality estimated from experimental data of trial 3: eave curtains and panels treated with *Beauveria bassiana*. Parameter values were chosen to minimise the residual sum of squares. *µ* is the mortality rate (per day), *β_s_* and *r_s_* are the dimensionless shape and rate shape parameters of the Weibull function, respectively, and *g* is the average time to death (in days) estimated from the Weibull function (see Additional file 1).

| Parameter | Control | Curtain | Panel |
| --- | --- | --- | --- |
| $\mu$ | 0.016 | 0.025 | 0.03 |
| $\beta_{s}$ | 3.4 | 8.9 | 8.5 |
| $r_{s}$ | 0.035 | 0.038 | 0.038 |
| *g* | 20.6 | 18.5 | 17.3 |
