# Supplementary material for: Exploiting the behaviour of wild malaria vectors to achieve high infection with fungal biocontrol agents
Source: Malar J. 2012 Mar 26;11:87. doi: 10.1186/1475-2875-11-87 (PMC3337815; doi:10.1186/1475-2875-11-87)
Supplement: Additional file 5 — Table S3 Parameters of the model of mosquito mortality estimated from experimental data of trial 4: eave baffles treated with Metarhizium anisopliae. Parameter values were chosen to minimise the residual sum of squares. Separate models were fitted for mosquitoes infected and uninfected with the fungus. μ is the mortality rate (per day), β, βs and r, rs are the dimensionless shape and rate shape parameters of the Weibull functions, respectively. g is the average time to death (in days) estimated from the Weibull function and gF is the estimated average time to death from the fungus infection alone (see Additional file 1). [file 1475-2875-11-87-S5.DOCX]

**Table S3.** Parameters of the model of mosquito mortality estimated from experimental data of trial 4: eave baffles treated with *Metarhizium anisopliae*. Parameter values were chosen to minimise the residual sum of squares. Separate models were fitted for mosquitoes infected and uninfected with the fungus. *µ* is the mortality rate (per day), *β*, *β_s_* and *r*, *r_s_* are the dimensionless shape and rate shape parameters of the Weibull functions, respectively. *g* is the average time to death (in days) estimated from the Weibull function and *g_F_* is the estimated average time to death from the fungus infection alone (see Additional file 1).

| Parameter | Control | Uninfected | Infected |
| --- | --- | --- | --- |
| $\mu$ | 0.011 | 0.016 | 0.016 |
| $\beta_{s}$ | 3.65 | 5.0 | 5.0 |
| $r_{s}$ | 0.039 | 0.036 | 0.036 |
| $\beta$ | 0 | 0 | 2.5 |
| $r$ | 0 | 0 | 0.067 |
| *g* | 20.3 | 20.65 | 11.5 |
| *g_F_* |  |  | 13.2 |
