# Supplementary material for: Exploiting the behaviour of wild malaria vectors to achieve high infection with fungal biocontrol agents
Source: Malar J. 2012 Mar 26;11:87. doi: 10.1186/1475-2875-11-87 (PMC3337815; doi:10.1186/1475-2875-11-87)
Supplement: Additional file 6 — Table S4 Parameters of the model of mosquito mortality estimated from experimental data of trial 4: eave baffles treated with Beauveria bassiana. Parameter values were chosen to minimise the residual sum of squares. Separate models were fitted for mosquitoes infected and uninfected with the fungus. Separate models were fitted for mosquitoes infected and uninfected with the fungus. μ is the mortality rate (per day), β, βs and r, rs are the dimensionless shape and rate shape parameters of the Weibull functions, respectively. g is the average time to death (in days) estimated from the Weibull function and gF is the estimated average time to death from the fungus infection alone (see Additional file 1). [file 1475-2875-11-87-S6.DOCX]

**Table S4.** Parameters of the model of mosquito mortality estimated from experimental data of trial 4: eave baffles treated with *Beauveria bassiana*. Parameter values were chosen to minimise the residual sum of squares. Separate models were fitted for mosquitoes infected and uninfected with the fungus. Separate models were fitted for mosquitoes infected and uninfected with the fungus. *µ* is the mortality rate (per day), *β*, *β_s_* and *r*, *r_s_* are the dimensionless shape and rate shape parameters of the Weibull functions, respectively. *g* is the average time to death (in days) estimated from the Weibull function and *g_F_* is the estimated average time to death from the fungus infection alone (see Additional file 1).

| Parameter | Control | Uninfected | Infected |
| --- | --- | --- | --- |
| $\mu$ | 0.011 | 0.026 | 0.026 |
| $\beta_{s}$ | 3.65 | 4.7 | 4.7 |
| $r_{s}$ | 0.039 | 0.037 | 0.037 |
| $\beta$ | 0 | 0 | 2.5 |
| $r$ | 0 | 0 | 0.069 |
| *g* | 20.3 | 17.9 | 10.45 |
| *g_F_* |  |  | 12.86 |
