# Supplementary material for: Exploiting the behaviour of wild malaria vectors to achieve high infection with fungal biocontrol agents
Source: Malar J. 2012 Mar 26;11:87. doi: 10.1186/1475-2875-11-87 (PMC3337815; doi:10.1186/1475-2875-11-87)
Supplement: Additional file 9 — Daily EIR as a function of the fungal biopesticide coverage. Line labels show the proportion of the mosquito population that is exophilic. [file 1475-2875-11-87-S9.DOCX]

Figure S1: Daily EIR as a function of the fungal biopesticide coverage. Line labels show the proportion of the mosquito population that is exophillic.
